# A Free Clinic and Does It Pay?

**Published:** 1914-04

**Authors:** Clarence A. Rhodes


### A FREE CLINIC AND DOES IT PAY?

By Clarence A. Rhodes, M. D., A. B.

It is a fact that can not be gain-said that the profession, at least that part of it who are engaged in pediatrics, have and are neglecting one of the most fertile fields in preventive and that which could be made the most progressive field of medicine in the south. We have reference to our factory or mill districts as they are called and of which Atlanta can boast of her full share.

The medical profession have not waked up to the importance of this field of work or at least have been slow in its development.

About two and a half years ago we took charge of the Wesley House Free Clinic for children, which is a department of an institutional institution called Wesley House Number One, which is located near the Fulton Bag and Cotton Mills. This clinic had been in existence for a number of years but had made no advancement; in fact it had gone to "the wall" when we took charge of it. There was no equipment at all, it being the custom of the physicians in charge to carry the equipment which they needed with them on their visits; this did not appeal to us so we made a rule that if those in charge of the institution could not furnish the necessary equipment for the clinic it had better be closed as the physician's time was enough for him to give when the clinic was a part of the institutional work. Working on this basis those in charge responded nobly and we soon had an equipment second to none in the city, and it is no boast to say far better than our medical schools and we are continually improving.

The staff at present consists of thirteen members, each a specialist in his line of work, having spent from one to six years in hospital work. No students are allowed to run the clinics and we have had to go slow even in allowing students to attend them, not that we are a closed combine and do not need the students' assistance, but on account of the class of patients we have to deal with. It is hard work to overcome their superstitions, pride and prejudices, and nothing will do this so quickly as gaining their confidence and by education both of which we try to accomplish, for this reason the clinics have not been opened to students, in fact, only two have applied and that recently so we are using them to a great advantage to the clinic and we hope of benefit to themselves, but it is difficult to make the patients realize the students are not experimenting on them but are there for their mutual benefit. However, in another year or two we hope to have their confidence and education up to such a standard that students will not be looked upon with suspicion but will be gladly received. It is our fondest hope that this time will speedily come, for we need the students' assistance in this work, and no better facilities are afforded in the city for their gaining knowledge than in the various departments of our clinic. The writer can speak especially for the children's clinic which is by far the best in the city from the stand-point of numbers and variety; the clinic was primarily intended for this work but the work has grown to such proportions that it was necessary to have a staff representing all the specialties. One of which we regret to say is not represented, but if some good philanthropic individual would open up his purse and equip this department it would be a God-send to those poor unfortunates; we have reference to a dental department which is badly needed but the funds are lacking, however, "Rome was not built in a day" so we are not discouraged, the dental department is bound to come.

Let it be understood, we do not consider our clinic an ideal by any means, as we have not the working force to make it so, but we hope to improve more and more as time goes on. You can see how impossible it is for one physician to take care of a large clinic with the assistance of one nurse and do all that is necessary to be done in order to bring it up-to-date in every respect. As an illustration we will use the children's clinic where we average from ten to fifteen and have had as high as forty at one clinic, so you see how impossible it is to take histories and keep records of the cases except mentally and a few notes which would be worthless for statistical purposes. The back-bone of the clinic is the excellent trained Christian nurse who is in charge all the time, and does the visiting in the neighborhood; the success of the clinic is largely due to her efforts.

The following statistics will give you a slight idea of the work that has been accomplished from Sept. 1st, 1912 to Dec. 31st, 1913, (sixteen months.)

Visits made by the nurse in the neighborhood 1,395.

Calls made on the nurse at the clinic not regular clinics, 3,343.

Clinics held, 273.

Patients treated at clinics, 2,468.

Prescriptions about 3000.

Dressings, 1,262.

Operations, 33.

Vaccinations, 237.

Treated for tape worm 4, for hook worm 11. It is surprising how few cases of hook worm have come under our observation while numerous examinations have been made; the reason for which is due to the crusade carried on in the district by the State Board of Health a few years ago.

Total number treated or rather treatments in the sixteen months 7,076, and average of 442 per month, one-fourth of this number being children or about 110 per month.

The amount of milk distributed and used by the clinic from June 15th, 1913 to January 1st, 1914, 500 gallons of butter milk and 375 gallons of sweet milk.

During the short time we have had charge of this work there have been marked changes wrought in the community and especially in the conditions of the patients, their intellects and habits. The mothers take more pride in their children; where they formerly brought them to the clinic with soiled hands and faces to say nothing about the condition of their clothes; now they bring them all "spruced up" to see "your doctor." It is well worth while when you see how devoted the little fellows become to the doctor and nurse, and during the whole clinic we do not have as much crying and fuss as you would have with one moderately spoiled child in a private family. The little ones being neglected at home are very responsive to any attention shown them. For instance, a chuckle under the chin in 90 per cent of the cases will elicit a smile, while a gentle pat or tickle on the "tummy" will bring forth a hearty laugh.

Dr. Henry L. Coit has given an excellent discription of tne class of people we are dealing with, they are termed the Devil's poor," "they are those who have reached poverty by their own deliberate transgression of physical and moral laws, they do not seem anxious to do better and are unable to take the iniative to escape from poverty without help. They are easily discouraged but with the judicious application of the principles of true charity, which stimulate effort and self-help, this class finally do well and make good. It pays to assist them and they and their families often become good and useful members of society." Is this not an excellent description of our "mill class" who are termed the "poor white trash" by our southern negro? Is it not a paying investment to better the conditions of these people?

There is just one word to be said in reference to the mothers of the district, and that is in one respect they are the best mothers on earth, for they all nurse their children and do not rob them of that God-given food which is customary with the mothers in the higher walks of life, and in consequence of this our death rate has been very low, only losing three babies during the last year, two being specifics and the other with whooping cough and broncho-pneumonia, all three having poor nursing at home. In speaking of the death rate we have reference to the children who have been regular attendants at our clinic and not to the whole mill district.

The work that is being done at the Wesley House will leave its imprint upon the future generations, and is it not a noble work to help our fellow man to a higher plane of living regardless of the "almighty dollar?"

